# Supplementary material for: Causal Relationship between Aging and Anorexia Nervosa: A White-Matter-Microstructure-Mediated Mendelian Randomization Analysis
Source: Biomedicines. 2024 Aug 16;12(8):1874. doi: 10.3390/biomedicines12081874 (PMC11351342; doi:10.3390/biomedicines12081874)
Supplement: Supplementary file 1 [file biomedicines-12-01874-s001.zip › Supplementary Figure/Figure S4/Singe SNP plot of MR analysis for TL to ubm-b-1935.pdf]

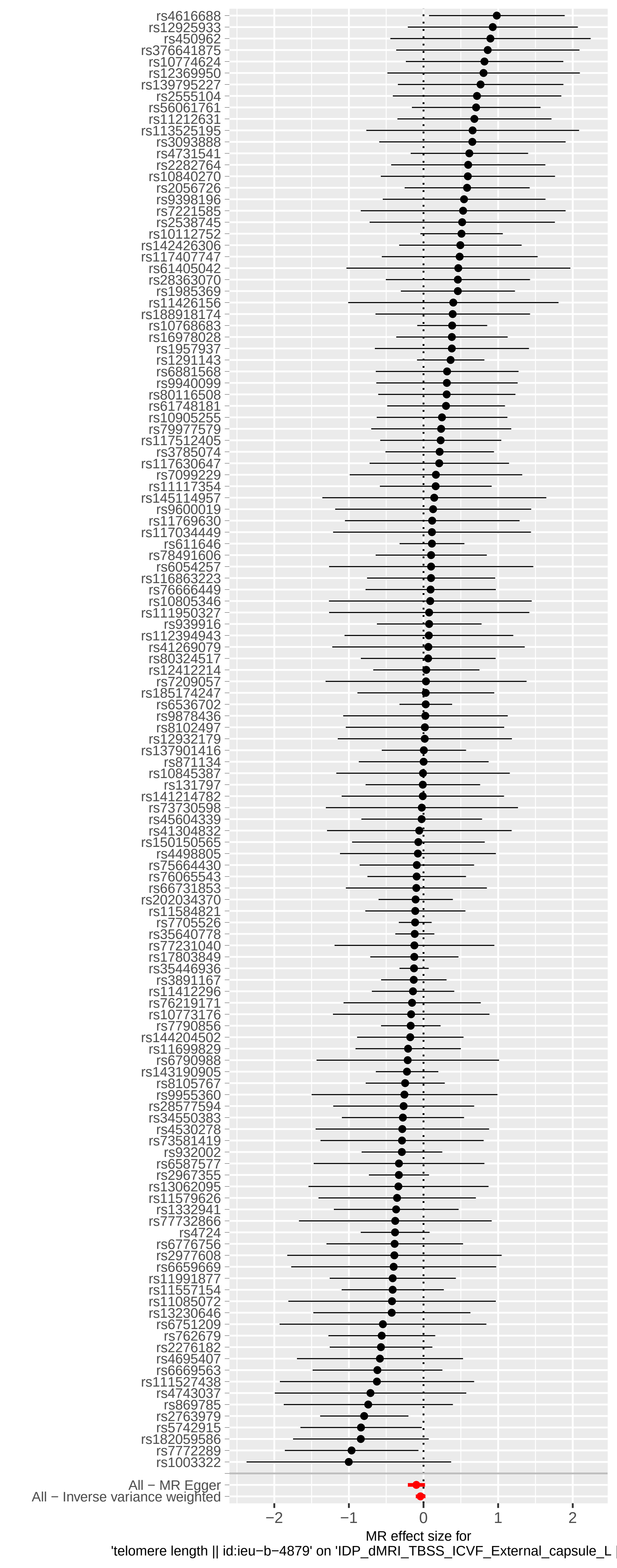
rs4616688
rs12925933
rs450962
rs376641875
rs10774624
rs12369950
rs139795227
rs2555104
rs56061761
rs11212631
rs113525195
rs3093888
rs4731541
rs2282764
rs10840270
rs2056726
rs9398196
rs7221585
rs2538745
rs10112752
rs142426306
rs117407747
rs61405042
rs28363070
rs1985369
rs11426156
rs188918174
rs10768683
rs16978028
rs1957937
rs1291143
rs6881568
rs9940099
rs80116508
rs61748181
rs10905255
rs79977579
rs117512405
rs3785074
rs117630647
rs7099229
rs11117354
rs145114957
rs9600019
rs11769630
rs117034449
rs611646
rs78491606
rs6054257
rs116863223
rs76666449
rs10805346
rs111950327
rs939916
rs112394943
rs41269079
rs80324517
rs12412214
rs7209057
rs185174247
rs6536702
rs9878436
rs8102497
rs12932179
rs137901416
rs871134
rs10845387
rs131797
rs141214782
rs73730598
rs45604339
rs41304832
rs150150565
rs4498805
rs75664430
rs76065543
rs66731853
rs202034370
rs11584821
rs7705526
rs35640778
rs77231040
rs17803849
rs35446936
rs3891167
rs11412296
rs76219171
rs10773176
rs7790856
rs144204502
rs11699829
rs6790988
rs143190905
rs8105767
rs9955360
rs28577594
rs34550383
rs4530278
rs73581419
rs932002
rs6587577
rs2967355
rs13062095
rs11579626
rs1332941
rs77732866
rs4724
rs6776756
rs2977608
rs6659669
rs11991877
rs11557154
rs11085072
rs13230646
rs6751209
rs762679
rs2276182
rs4695407
rs6669563
rs111527438
rs4743037
rs869785
rs2763979
rs5742915
rs182059586
rs7772289
rs1003322
All − MR Egger
All − Inverse variance weighted
−2
−1
0
1
2
MR effect size for
'telomere length || id:ieu−b−4879' on 'IDP_dMRI_TBSS_ICVF_External_capsule_L |
